# Supplementary material for: Motor axonopathies in a mouse model of Duchenne muscular dystrophy
Source: Sci Rep. 2020 Jun 2;10:8967. doi: 10.1038/s41598-020-65824-1 (PMC7265344; doi:10.1038/s41598-020-65824-1)
Supplement: Supplementary file 1 — Supplementary information. [file 41598_2020_65824_MOESM1_ESM.pdf]

Motor axonopathies in a mouse model of Duchenne muscular dystrophy

Justin S. Dhindsa[1], Angela L. McCall[1], Laura M. Strickland[1], Anna F. Fusco[1], Amanda F. Kahn[1], Mai K. Elmallah[1*]

[1] Division of Allergy, Immunology, and Pulmonary Medicine, Department of Pediatrics, Duke University Medical Center Box 2644, Durham, North Carolina 27710, USA

*Correspondence: mai.elmallah@duke.edu

Supplementary Figures

Supplemental Figure Titles and Legends

Figure S1 Small-caliber axons are not demyelinated in *mdx* mice

a, Myelin sheath thickness of small-caliber axons (< 5 μm) in wildtype mice (phrenic: 1.94 μm ± 0.06, *n* = 45 axons; XII: 1.86 μm ± 0.02, *n* = 124 axons) and *mdx* mice (phrenic: 2.00 μm ± 0.02, *n* = 105 axons; XII: 1.82 μm ± 0.01, *n* = 229 axons). Phrenic: *p* = 0.234 Two Sample *t*-test; XII: *p* = 0.170 Two Sample *t*-test.

Figure S2 Phrenic and XII motor neuron counts

a, Bright field images of C4 spinal cord sections. Dashed circle around the phrenic motor pool. **b,** Bright field images of medulla sections. Scale bars represent 100 μm. **c,** Average phrenic motor neurons per cervical spinal cord section (Wildtype: 11.87 motor neurons ± 0.58, *n* = 2 mice; *mdx:* 14.51 motor neurons ± 1.71, *n* = 3 mice). **d,** Average XII motor neurons per medulla section (Wildtype: 136.01 motor neurons ± 11.20, *n* = 3 mice ; *mdx:* 117.10 motor neurons ± 5.08, *n* = 3 mice). Phrenic: *p* = 0.261 Two Sample *t*-test; XII: *p* = 0.229 Two Sample *t*-test.

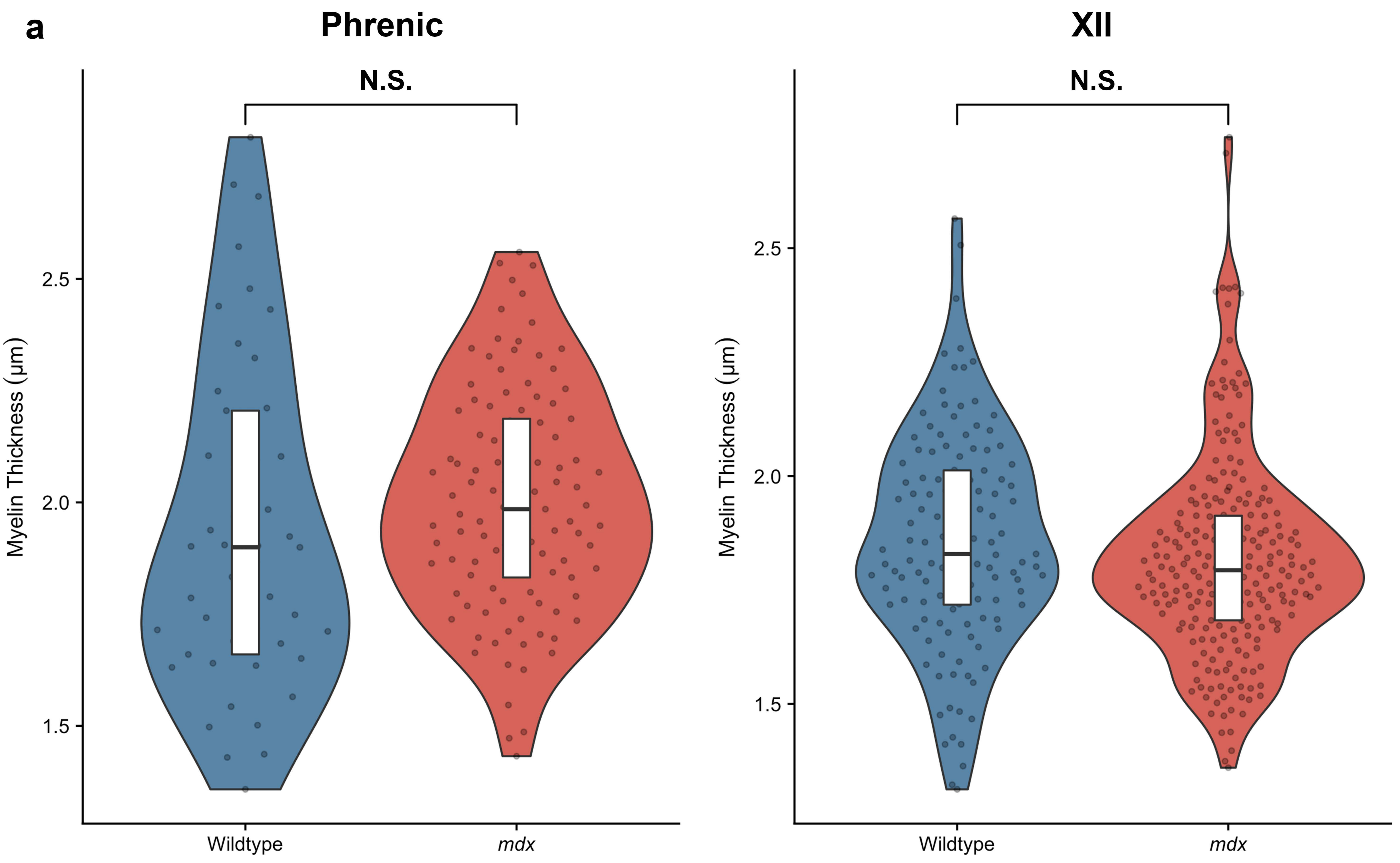
a
Phrenic
XII
N.S.
N.S.
Myelin Thickness (μm)
Myelin Thickness (μm)
2.5
2.0
1.5
2.5
2.0
1.5
Wildtype
mdx
Wildtype
mdx

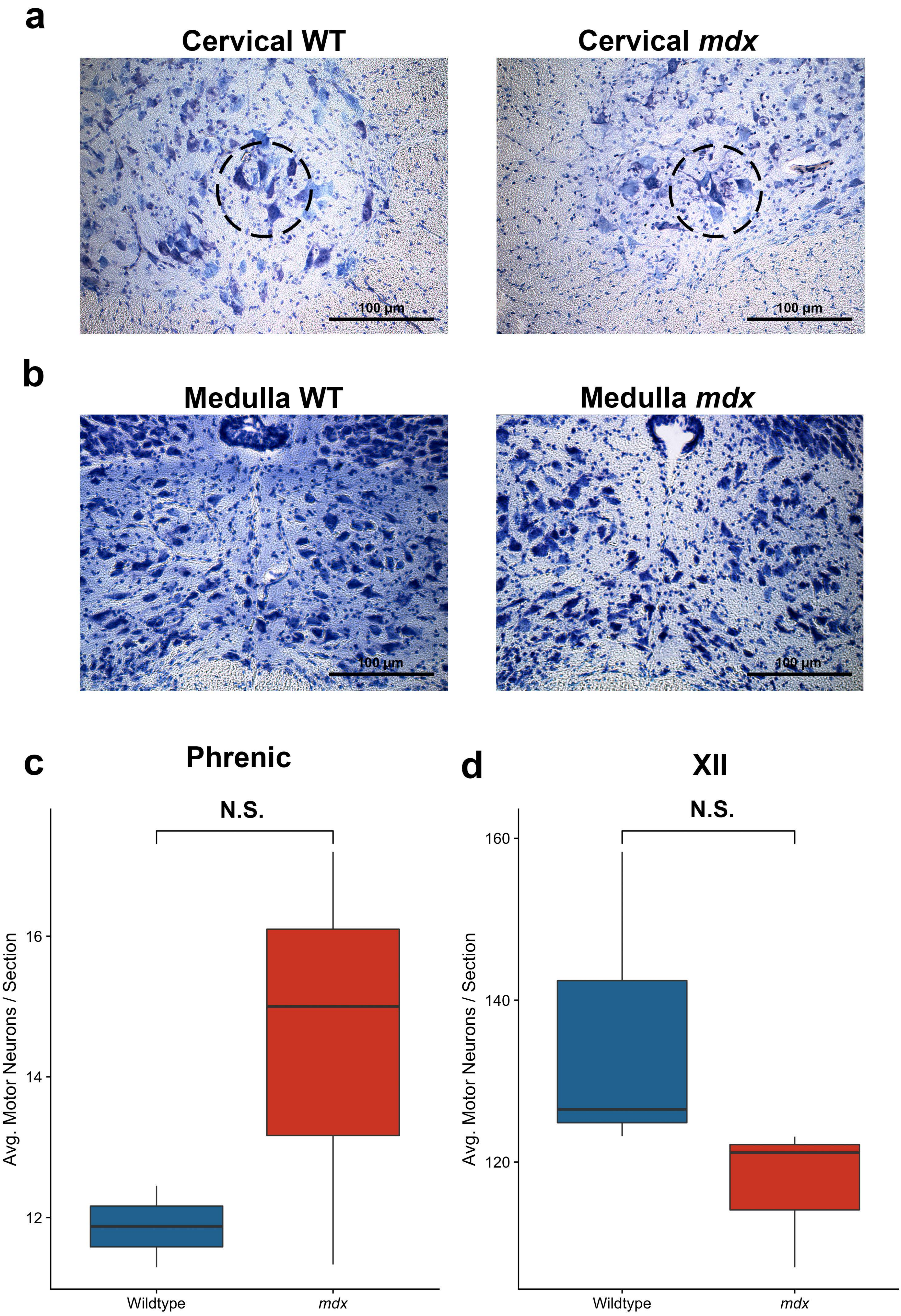

a
Cervical WT
Cervical mdx
100 μm
100 μm
b
Medulla WT
Medulla mdx
100 μm
100 μm
c
Phrenic
N.S.
Avg. Motor Neurons / Section
12
14
16
Wildtype
mdx
d
XII
N.S.
Avg. Motor Neurons / Section
120
140
160
Wildtype
mdx
